# Mechanisms and Determinants of CMV Reactivation in Kidney Transplantation

**DOI:** 10.3390/ijms27156727

**Published:** 2026-07-28

**Authors:** Ruchi Naik, Walaa Dabbas, Benito Veldepenas, Demetrius Harvell, Fares Eshac, Megan Trivedi, Carlo Minicucci, Mary Hummel, Zheng Jenny Zhang, Lorenzo Gallon, Eleonora Forte

**Affiliations:** 1 Division of Nephrology, Department of Medicine, University of Illinois College of Medicine, Chicago, IL 60612, USA; 2 Division of Transplantation, Department of Surgery, University of Illinois Chicago, Chicago, IL 60612, USA; 3 Department of Pharmacy Practice, University of Illinois Chicago, Chicago, IL 60612, USA; 4 Department of Surgery, Northwestern University Feinberg School of Medicine, Chicago, IL 60611, USA

**Keywords:** CMV, reactivation, kidney transplant, inflammation, epigenetics, immunosuppression

## Abstract

Human cytomegalovirus (CMV) remains a significant infectious complication after kidney transplantation, reflecting gaps in the understanding of the molecular and immunological mechanisms regulating the transition from latency to productive infection. Following primary infection, CMV establishes lifelong latency in hematopoietic and myeloid lineage cells, maintained by viral chromatin repression and robust CMV-specific immune surveillance. CMV reactivation is associated with graft dysfunction, increased risk of rejection, opportunistic infections, and reduced patient survival. In kidney transplantation, CMV reactivation is driven by the interplay between tissue injury, inflammation, and immunosuppression. Ischemia–reperfusion injury and peri-operative stress produce reactive oxygen species, DNA damage, and pro-inflammatory cytokines (e.g., TNF-α, IL-6), which activate transcription factors such as NF-κB and AP-1. These factors regulate the CMV major immediate-early promoter (MIEP), thereby triggering lytic viral gene expression. At the same time, immunosuppressive therapies impair antiviral immune surveillance and, in some cases, induce cytokine release, potentially contributing to the pro-inflammatory environment that favors viral reactivation. In this review, we summarize current molecular and immunologic mechanisms governing CMV latency and reactivation with a focus on how immunosuppressive strategies and injury-associated pathways converge to promote CMV reactivation. We also discuss implications of risk stratification and the development of targeted therapeutic strategies to prevent CMV reactivation in kidney transplant recipients (KTRs).

## 1. Introduction

Human cytomegalovirus (CMV) is a β-herpesvirus that infects up to 90% of people worldwide and establishes a lifelong infection in the host through a regulated balance between latent and lytic infection, which is controlled by host immune surveillance and epigenetic regulation of the viral genome [1]. Human CMV is a double-stranded linear DNA virus with the largest genome among human viruses, with a size of approximately 236 kb. The complex genome encodes more than 200 open reading frames, 4 major long noncoding RNAs, and 26 mature microRNAs (miRNAs). The genome is organized into unique long (UL) and unique short (US) regions flanked by repeated sequences, and encodes viral products involved in replication, immune evasion, latency, and reactivation [2,3].

During latency, viral gene expression is limited, allowing CMV to evade immune detection while persisting in multiple host cell types, including hematopoietic progenitor cells (HPCs), myeloid lineage cells, and endothelial cells. Under specific conditions, this balance can be disrupted, leading to viral reactivation and productive infection, thereby enabling viral transmission to other hosts [4,5,6].

While our understanding of CMV latency continues to evolve, latency is generally defined as a phase in which the viral genome persists in the nucleus of the infected cell in an episomal form, with a limited viral gene expression and absence of productive viral replication. Reactivation refers to the transition to a productive (lytic) phase characterized by increased viral gene expression, viral DNA replication, and the production of infectious virions [7].

In immunocompetent individuals, the immune system keeps CMV infection in check and clears actively infected cells, resulting in a mild or asymptomatic infection. However, latently infected cells are not eradicated by the immune system, and viral reactivation may lead to disease, especially in immunocompromised individuals. CMV infection remains a significant threat to kidney transplant recipients (KTRs), causing significant morbidity and mortality [8].

In kidney transplantation, the risk of CMV infection and disease is primarily determined by the donor and recipient CMV serostatus. CMV-seronegative recipients receiving organs from CMV-seropositive donors (D+/R−) represent the highest-risk group because exposure to donor-derived latent CMV occurs in the absence of pre-existing CMV-specific immunity. CMV-seropositive recipients (D+/R+ or D−/R+) have intermediate risk, as viral replication generally results from reactivation of a previously acquired latent CMV infection, although immunosuppression can impair immune control, allowing viral replication. CMV-seronegative recipients receiving organs from seronegative donors (D−/R−) have the lowest risk but remain susceptible to primary infection through community exposure or transfusion. Throughout this review, CMV infection refers to the detection of CMV replication, whereas CMV disease refers to symptomatic or tissue-invasive infection. CMV reactivation specifically refers to renewed viral replication from a latent state.

In KTR, CMV reactivation after transplantation is driven by the combination of surgery-induced tissue injury, inflammation, and chronic immunosuppression, which together facilitate viral replication, leading to viremia and CMV-associated diseases. These processes are associated with the release of pro-inflammatory cytokines that can activate transcriptional signaling pathways implicated in reactivation. Clinically, CMV reactivation is associated with a higher risk for acute and chronic rejection and diminished graft survival [9,10,11].

CMV infection ranges from asymptomatic viremia to severe tissue-invasive disease. The most common clinical presentation is CMV syndrome, characterized by fever, malaise, leucopenia, thrombocytopenia, and elevated liver enzymes. In more severe cases, CMV disease may involve the gastrointestinal tract, lungs, liver, retina, central nervous system, or renal allograft. Beyond direct tissue injury, CMV infection exerts indirect immunomodulatory effects promoting systemic inflammation, endothelial dysfunction, and activation of alloimmune pathways, thereby increasing the risk of rejection and chronic allograft injury. Additionally, CMV infection can directly involve the renal allograft, producing lesions such as tubulointerstitial nephritis, glomerulopathy, or vascular injury that contribute to graft dysfunction and potential graft loss [12]. CMV has also been associated with secondary opportunistic infections and increased mortality.

Although antiviral prophylaxis and preemptive strategies have significantly reduced early CMV disease, they do not eliminate latent infection or fully prevent reactivation. Consequently, CMV infection remains common in the KTRs, with a reported incidence rate approaching 40% in some cohorts [13]. In addition, antiviral therapies are often associated with toxicity and the risk of drug resistance, and no effective vaccine is currently available [14,15,16].

Together, these limitations underscore the need for a deeper understanding of the molecular and immunologic mechanisms governing CMV latency and reactivation, particularly in the context of immunosuppressive therapy.

## 2. Molecular and Cellular Mechanisms Underlying CMV Latency and Reactivation

### 2.1. Models of CMV Latency and Reactivation

Several experimental models have been used to study the mechanisms underlying CMV latency and reactivation, both in cellular and animal systems. While no single model fully captures all aspects of CMV infection, the available systems provide complementary insights into the mechanisms that regulate latency, reactivation, and productive infection.

#### 2.1.1. Human In Vitro Models of HCMV Latency and Reactivation

In vitro models of latency are mainly based on human myeloid cells. Primary CD34^+^ HPCs are considered the gold standard model in the field, as latency is naturally established in these cells [17,18]. However, their use is limited by donor heterogeneity, low availability, high cost, and low reactivation efficiency. To address these limitations, human embryonic stem cell-derived (hESC) CD34+ cell models have recently been developed [19]. In these cells, reactivation typically occurs in conjunction with myeloid differentiation into macrophages and dendritic cells and is commonly induced by co-culture on a stromal monolayer in the presence of pro-inflammatory cytokines and growth factors [20]. Beyond hESC-derived CD34+ cells, induced pluripotent stem cells (iPSCs) have also emerged as valuable systems for modeling CMV latency and reactivation. iPSCs derived from myeloid cells exhibit many of the key features of primary myeloid cells, offering advantages such as ease of genetic modification, controlled differentiation, and unlimited availability [21].

Primary CD14+ peripheral blood mononuclear cells are another recognized site of latency and have also been widely used as an experimental model for latency and reactivation, with viral reactivation similarly associated with differentiation, specifically to M1 proinflammatory macrophages [22,23].

Myeloid cell lines, including monocytic THP-1 and the myeloid progenitor Kasumi-3 cells, have also been extensively used to investigate HCMV latency and reactivation. Relative to primary cells, these immortalized cell lines offer several advantages. They are inexpensive, easy to maintain, and can be cultured in large numbers; however, because they are immortalized, they exhibit dysregulated signaling and do not fully recapitulate the physiological latency and reactivation that occur in vivo [24].

In general, all in vitro models of CMV latency and reactivation have several limitations, including rapid loss of viral DNA, which constrains studies in primary cells with limited material, and incomplete silencing of viral gene expression [25]. In addition, these cell-based model systems lack the complex immune and tissue environments that, in vivo, contribute to latency and reactivation. Therefore, although these models provide valuable information, their results cannot fully recapitulate what occurs in vivo.

#### 2.1.2. Murine In Vivo Models of MCMV Latency and Reactivation

HCMV does not naturally infect mice; therefore, murine CMV (MCMV) is widely used as a valid surrogate for studying CMV biology due to its similarities to HCMV, including structure/function, ability to establish latency and to reactivate, cell tropism, and regulatory elements, which are extensively reviewed [1,26,27,28]. Studies using MCMV models have demonstrated tissue- and cell-type-specific reservoirs of latent infection, providing information difficult to obtain from in vitro models. In the kidney and liver, endothelial cells represent a major site harboring latent viral genomes, whereas in the lung and bone marrow, latency is primarily established within myeloid lineage cells [22,29].

### 2.2. Mechanisms Underlying CMV Latency Establishment and Maintenance

Following primary infection, CMV infects permissive cell types, including fibroblasts, endothelial cells, and epithelial cells, facilitating viral dissemination. These infected cells promote the dissemination of CMV to hematopoietic progenitor cells (CD34+) and myeloid cells (CD14+ monocytes), where latency is established in the absence of active viral replication. These myeloid cells serve as long-term reservoirs and play a central role in viral persistence and reactivation [30,31,32]. In addition to myeloid cells, endothelial cells have been reported as a site of viral persistence [29,33].

Molecular mechanisms underlying latency are not fully defined and can be cell-type dependent; however, it is well established that epigenetic regulation of viral chromatin and tightly controlled viral gene expression play central roles in the establishment and maintenance of latency [34].

Viral DNA is delivered to host cells in a naked state. Upon nuclear entry, viral DNA rapidly associates with host histones and becomes subject to epigenetic regulation [35]. Intrinsic antiviral promyelocytic leukemia nuclear bodies (PML-NBs) associate with incoming viral DNA and recruit chromatin-modifying proteins, including PML, death domain-associated protein (hDaxx), (ATRX), and Sp100, to promote heterochromatinization [36,37].

The viral major immediate-early promoter (MIEP), which controls the expression of immediate-early proteins IE1 and IE2, functions as the key regulator of the lytic transcriptional cascade. IE proteins initiate the expression of Early (E) genes involved in viral DNA replication, followed by the expression of Late (L) genes encoding structural proteins required for viral particle assembly.

During latency, this transcriptional program is inhibited by a multilevel regulation of the MIEP, including chromatin accessibility, histone modifications, and transcription factor binding. During latency, MIEP is associated with repressive chromatin modifications such as methylation of histone H3 at lysine 9 (H3K9) and lysine 27 (H3K27), which inhibit viral lytic gene expression [38,39].

Several chromatin-associated factors participate in establishing and maintaining MIEP repression, including heterochromatin protein 1 (HP1) [40], Ying Yang 1 (YY1) [41], the polycomb repressive complex 2 (PRC2) [42], KRAB-associated protein 1 (KAP1) [43], CCCTC-binding protein (CTCF) [44]. Histone deacetylases (HDACs) further contribute to MIEP transcriptional repression by removing activating acetylation marks and maintaining a closed chromatin conformation at the MIEP [40]. Together, these factors promote a repressive chromatin profile through histone methylation, histone deacetylation, and chromatin looping. Despite the repression of viral transcription during latency, latency is not entirely transcriptionally silent. A subset of viral genes, including latency-associated transcripts such as latency unique natural antigen (LUNA) and UL138, is expressed and contributes to the maintenance of latency, although they are not exclusively restricted to this phase and can also be detected during lytic infection [45].

Viral miRNAs are also expressed during latency and contribute to latency establishment and maintenance by regulating both viral and host gene expression. For example, miR-UL112-3p directly targets the immediate-early protein IE1, thereby inhibiting lytic replication [46,47]. Other miRNAs contribute to immune evasion [48], limit the proliferation of latently infected cells, and restrain myeloid cell differentiation, a process linked to reactivation [49].

New evidence suggests that the latency-associated transcription profile resembles the lytic one, albeit at reduced levels [50], suggesting that viral gene expression is regulated not only by epigenetic constraints but also by context-dependent signaling pathways to control latency and active transcription programs. For example, some host transcription factors that regulate viral transcription during active CMV infection may be absent or inactive during latency but become available in response to inflammatory or differentiation signals [1,45].

The expression levels of these genes decline over time, concomitant with transcriptional repression observed during latency, consistent with a model in which latency is established following transient activation of viral gene expression, which is first turned on and then repressed [24]. This is supported by findings in myeloid progenitor cells demonstrating dynamic chromatin remodeling and early activation of viral gene expression, prior to the establishment of transcription repression [51]. Despite this transient transcriptional activity, latency-associated viral gene expression remains below the threshold required to trigger a robust host antiviral immune response, thereby facilitating immune evasion and the persistence of CMV infection.

In summary, latency is maintained through multilayered epigenetic repression of MIEP, while reactivation requires coordinated chromatin remodeling and transcription factor recruitment. Importantly, HCMV infection extensively alters both viral and host chromatin organization, creating a transcriptional environment that supports either latent persistence or lytic infection [52,53].

### 2.3. Molecular Mechanisms of CMV Reactivation

CMV reactivation is triggered when the epigenetic and transcriptional mechanisms that maintain latency are disrupted by inflammatory signaling, myeloid cell differentiation into dendritic cells, DNA damage, and tissue injury.

The MIEP functions as a key molecular switch between latency and reactivation. During reactivation, repressive epigenetic marks are removed from the MIEP, allowing its de-repression, immediate early gene expression, and activation of the lytic transcriptional cascade.

In reactivated cells, the MIEP adopts an open chromatin structure characterized by activation marks, including acetylated histone H4, and loss of HP1 binding, resulting in increased chromatin accessibility and transcriptional activation [38,54,55].

MIEP de-repression is orchestrated by both viral and host factors. For example, the viral tegument protein pp71 restores MIEP activity by downregulating the repressive histone H3K27 trimethylation through ubiquitin-mediated degradation of the methyltransferase EZH2, a core component of PRC2 [56].

The host KRAB-associated protein 1 (KAP1) also contributes to differential regulation of the MIEP during latency and reactivation in response to myeloid cell differentiation. Specifically, during latency, KAP1 recruits the heterochromatin-inducing factors HP1 and the H3K9me3 histone methyltransferase SET domain, bifurcated 1 (SETDB1) to viral promoters, including the MIEP, in a CD34+ cell in vitro model. After their differentiation into mature dendritic cells, KAP1 becomes phosphorylated, impairing its ability to recruit repressor factors to viral chromatin and thereby derepressing the MIEP, which then becomes accessible to host transcription factors that activate lytic viral gene expression [43].

The MIEP contains binding sites for a variety of transcription factors, including activators such as Nuclear Factor kappa-light-chain-enhancer of activated B cells (NF-κB) and activator protein 1 (AP-1), which activate viral gene expression and recruit epigenetic factors, such as histone acetyltransferases, to the promoter to enable activation. Activator transcription factors are regulated by upstream signaling, including inflammation, DNA damage, and injury [1].

Inflammatory cytokines, such as Tumor Necrosis Factor (TNF-α) and Interleukin-6 (IL-6), can reactivate the MIEP via NF-κB in vitro [57,58,59,60]. This is particularly relevant in the context of transplantation, as the surgery itself induces the release of inflammatory mediators that contribute to CMV reactivation. In transplantation, inflammation, which promotes viral gene expression, and immunosuppression, which impairs the anti-CMV immune response, act synergistically, facilitating viral reactivation and dissemination [28,61,62,63].

Differentiation of latently infected CD34+ HPCs or monocytes into macrophages or dendritic cells is a well-established mechanism that promotes CMV reactivation. This differentiation creates a cellular environment permissive for IE gene expression through chromatin remodeling, loss of repressor factors expressed in undifferentiated cells, and induction of transcription factors that activate the MIEP [1,55,64,65,66,67]. Importantly, CMV infection modulates monocyte differentiation by promoting an M1/M2 macrophage phenotype that facilitates viral persistence, increased motility, and viral dissemination [6]. This was also confirmed in a murine system, in which CMV infection was shown to remodel the identity of CMV-infected bone marrow (BM)-derived macrophages, which exhibited markedly altered morphology, loss of macrophage-specific surface markers, and acquisition of a more stem-cell-like phenotype. In addition, these cells exhibited impaired antigen-presenting ability and increased migration and invasiveness, thereby facilitating viral dissemination [68].

Notably, although myeloid differentiation is a major mechanism of CMV reactivation, reactivation can also occur independently of myeloid differentiation, as shown in the myeloid progenitor Kasumi-3 cell model, which is refractory to differentiation but can be reactivated by inflammatory stimuli such as TNF-α [24].

Viral proteins also modulate the latency/reactivation switch. One example is LUNA, which is expressed in both phases of the viral life cycle, performing distinct roles. During latency, LUNA modulates chromatin configuration, enhancing the association of the myeloid transcription factor GATA-binding factor 2 (GATA2) with latency promoters and thereby supporting the transcription of latency-associated genes. During reactivation, LUNA disrupts antiviral PML-NBs through its deSUMOylation activity, promoting an environment that supports lytic gene expression [69].

Another important viral regulator is the UL133-UL138 gene locus, encoding the viral proteins UL133, UL135, UL136, and UL138 [70]. Among these proteins, UL138 and UL135 are particularly relevant because they antagonistically regulate latency and reactivation through the modulation of host signaling pathways. Specifically, these viral proteins antagonistically regulate the epidermal growth factor receptor (EGFR) signaling pathway, a critical upstream regulatory node that regulates downstream signaling and trafficking pathways governing the survival, motility, and differentiation of infected monocytes and CD34+ HPCs, processes that enable viral latency, persistence and subsequent CMV reactivation [71]. UL135 has been shown to inhibit EGFR by promoting its removal from the cell surface and subsequent degradation, enabling reactivation, whereas UL138 maintains EGFR at the cell surface, favoring latency [72,73].

One of the main downstream pathways of EGFR is the mitogen-activated protein kinase (MAPK) signaling pathway. In CD34+ HPCs, MAPK signaling has been shown to support CMV latency by inducing Early Growth Response 1 (EGR-1) expression, which in turn promotes UL138 expression [70]. Accordingly, pharmacological inhibition of MAPK signaling has been associated with CMV reactivation in Kasumi-3 cells [74].

In contrast, during inflammatory stimulation of dendritic cells, IL-6-induced MAPK signaling, together with Src family kinase (SFK) signaling, promotes chromatin remodeling at the MIEP, facilitating viral gene expression and viral reactivation [67,75].

Collectively, viral regulatory mechanisms, inflammatory signaling pathways, and cellular differentiation act on the MIEP to establish a transcriptionally permissive environment that supports lytic viral gene expression and CMV reactivation.

### 2.4. Murine Kidney Transplantation Models of CMV Reactivation

Murine kidney transplantation models have been developed in which transplantation can be performed across both major histocompatibility complex (MHC)-matched and MHC-mismatched strains. This experimental flexibility enables mechanistic separation of ischemia–reperfusion injury (IRI) from alloimmune responses in driving CMV reactivation—an approach that is not feasible in clinical transplantation. In high-risk D^+^/R^−^ kidney transplant models, transplantation induces expression of the immediate-early genes IE1 and IE3 within two days post-transplantation. This early activation coincides with the induction of inflammatory signaling pathways and transcription factors such as NF-κB and AP-1, which are classically associated with transplant-related IRI. These transcription factors bind the MIEP during acute infection and early post-transplantation, but their occupancy is lost as latency is established, concomitant with recruitment of transcriptional repressors and silencing of IE gene expression [63,76,77,78,79].

Accumulating evidence identifies transplant-associated IRI as a key trigger of CMV reactivation. Notably, viral reactivation and dissemination occur in syngeneic kidney transplants in a manner comparable to allogeneic transplants, indicating that IRI alone, independent of alloimmune mismatch, is sufficient to initiate reactivation. In contrast, treatment of latently infected mice with clinically relevant immunosuppressive regimens in the absence of transplantation does not induce viral reactivation. Furthermore, activation of cellular stress pathways correlates with CMV reactivation, while donor-specific tolerogenic strategies that dampen alloimmune inflammation fail to prevent reactivation [80]. Importantly, CMV infection itself can disrupt established transplant tolerance [81].

Together, these findings support a “two-hit” model of CMV reactivation in which IRI associated with graft implantation constitutes the first hit, initiating transcriptional reactivation of latent virus, whereas immunosuppression serves as the second hit, enabling viral dissemination and productive infection [82]. Further elucidation of the cellular and molecular mechanisms underlying the initial IRI-driven trigger is essential for developing strategies to prevent CMV reactivation while simultaneously mitigating transplant-associated injury [83].

## 3. Immune Control of CMV Infection and Mechanisms of Viral Immune Evasion

### 3.1. Adaptive Immune Surveillance in Active CMV Infection: CD8+ and CD4+ T Cells

In immunocompetent individuals, the host responds to HCMV infection with robust cellular immunity, characterized by the expansion of CMV-specific CD8+ cytotoxic T cells, along with supportive CD4+ T-cell responses.

CMV-specific CD8+ T cells, which are mainly directed against immunodominant viral antigens such as pp65 and IE1, exhibit an advanced effector memory differentiation state and accumulate over time rather than contracting after the acute phase of infection, a phenomenon known as memory inflation [84,85].

Repeated antigenic stimulation can further amplify memory inflation, contributing to the accumulation of CMV-specific CD8+ T cells over time [86,87]. These cells are generally highly differentiated (e.g., CD45RA+, CD57+, CD28-) and exhibit a transcriptional effector profile characterized by the expression of T-bet, EOMES, and IFN-related genes, granzyme B, perforin, and CX3CR1 [87,88]. Notably, CMV-specific CD8+ T cells retain long-term intrinsic cytolytic properties, unlike cells in other chronic viral infections, which typically exhibit an exhausted phenotype [87].

CMV-specific CD8+ T-cells are present at high frequencies; however, their maintenance and function depend on CD4+ T-cell help. CMV-specific CD4+ T cells help sustain CD8+ T-cell responses by promoting dendritic cell licensing through CD40-CD40L interactions. CD4+ T cells recognize viral peptides presented by MHC class II molecules and provide activating signals to dendritic cells. This process upregulates the costimulatory molecules CD80 and CD86, as well as promotes antigen presentation and the secretion of chemokines such as CCL3 (C-C Motif Chemokine Ligand 3) and CCL4 (C-C Motif Chemokine Ligand 4). These signals facilitate the interaction between CD8+ T cells and licensed dendritic cells, supporting effective CD8+ priming and long-term maintenance [89,90,91].

In immunosuppressed solid-organ transplant recipients, delayed or impaired CMV-specific CD4+ T-cell responses are associated with prolonged viremia and a higher risk of CMV disease [92]. Restoration of functional CMV-specific CD4+ T cells supports immune recovery and protection against CMV reactivation in CMV-seropositive recipients, indicating that effective CMV control requires both CMV-specific CD4+ and CD8+ T-cell immunity. Consistent with this, in the absence of CMV-specific CD4+ T cells, CMV-specific CD8+ cytotoxicity can transiently recover but later declines, indicating that CD4+ T cells are important for long-term CD8+ T-cells- mediated viral control [93,94,95].

In addition to their supporting role, CMV+-specific CD4+ T cells may also contribute directly to antiviral protection. Higher pre-transplant CMV-specific CD4+ T cell responses are associated with faster post-transplant immune recovery and fewer CMV infections requiring antiviral treatment [96]. In addition, in some CMV-seropositive KTRs, CMV-specific CD4+ T cells together with CMV-specific antibody-producing B cells were still able to provide sufficient protection against reactivation despite minimal or undetectable CD8+ T-cell frequencies, suggesting that CD4+ T-cells can exert antiviral protection even when CD8+ T cell responses are limited. CMV-specific CD4+ T cells can also exert direct antiviral effector functions through cytotoxic mechanisms, including granzyme B- and perforin 1- mediated killing, and by producing antiviral cytokines such as interferon-γ (IFN-γ) [97]. These cells exhibit a cytotoxic phenotype driven primarily by CMV infection itself, rather than by kidney transplantation or immunosuppressive interventions [98].

### 3.2. Innate Immune Surveillance: Natural Killer (NK) Cells

Innate immune cells, particularly NK cells, play a key role in controlling CMV infection during the early phases of active infection and viral reactivation. During the early innate response, pathogen-associated molecular patterns (PAMPs) are recognized by pattern recognition receptors (PRRs) expressed by infected cells and immune cells, including dendritic cells, macrophages, and monocytes. This activates downstream signaling pathways that activate NK cells, leading to the release of pro-inflammatory cytokines, including IFN-γ and TNF-α, and the elimination of infected cells through cytotoxic activity, thereby limiting viral replication and dissemination during the early stages of infection and reactivation [99,100].

Following CMV primary infection, NK cells expand and differentiate into adaptive (memory-like) NK cell populations that are characterized by the expression of markers such as natural killer group 2C (NKG2C), CD57, and inhibitory killer cell immunoglobulin–like receptors (KIRs) [101,102,103]. Adaptive NKG2C+ NK cells preferentially expand in CMV-seropositive solid-organ transplant recipients and undergo remodeling during episodes of post-transplant CMV viremia [104].

In CMV-seropositive KTRs, CMV reactivation has been linked to the expansion of an adaptive NKG2C+ NK cell population expressing CD56 and increased CD107a/LAMP-1, a degranulation marker consistent with heightened cytotoxic activity, together with a reduction in the CD16^+^ NKG2A^−^ CD57^+^ subset [105]. The expansion of NKG2C+ NK cells is a characteristic feature of CMV infection, observed in the transplant setting and in healthy CMV-seropositive individuals, whereas it is rare in CMV-seronegative recipients [105]. However, the magnitude of the expansion appears greater in immunocompromised patients, likely due to a higher viral burden and less effective T-cell control [106].

Expansion of NKG2C+ NK cells has been associated with higher viral loads at diagnosis, suggesting that their expansion reflects antigenic burden during clinically detectable CMV replication. After viremia resolution, adaptive NKG2C+ NK cells with memory-like features remain increased, consistent with durable imprinting of the NK compartment even after viral control is restored [101,105].

### 3.3. Viral Immune Evasion in Latently Infected Cells

Transcriptional silencing of the MIEP alone cannot fully explain the long-term persistence of CMV in latently infected cells. During latency, CMV employs multiple immune evasion strategies to counter host immune surveillance, limit immune recognition, and promote viral persistence. Latency-associated products, including several viral proteins and microRNAs, are known to limit antigen presentation, dampen interferon signaling, and control NK cell activation. For example, miR-UL112-1 targets MHC-I-related chain B (MICB), a ligand of natural killer group 2D (NKG2D), an activating NK receptor implicated in the elimination of infected cells. MICB repression reduces NK-cell recognition and inhibits NK-mediated cytotoxicity [107]. The same miRNA also targets the IE1 transcript itself to prevent T cell recognition of latently infected cells [108]. In addition to hijacking immune recognition, viral miRNAs during latency also limit the production of inflammatory cytokines. miR-UL148D, for example, was shown to restrict secretion of IL-6 by targeting Activin A Receptor Type 1B (ACVR1B) [109]. Additionally, CMV encodes homologs of host cytokines to manipulate the immune response for its benefit. UL111A, for example, encodes different isoforms of viral IL-10 homolog, which are differentially expressed during latency and productive infection. These viral IL-10 isoforms have been shown to suppress pro-inflammatory cytokine production and impair antigen-presenting cell functions [108]. Other miRNA-mediated strategies contributing to immune evasion and HCMV latency have been reviewed elsewhere [48].

### 3.4. Viral Immune Evasion in Actively Infected Cells

During active CMV infection, a variety of immune evasion strategies are deployed to evade the host immune response, including impairing the differentiation of monocytes into dendritic cells, thereby disrupting the function of antigen-presenting cells. CMV infection can reduce the expression of MHC classes I and II molecules on the cell surface and diminish essential costimulatory signals such as CD40 and CD80 [110]. A significant mechanism underlying MHC class II downregulation involves the mRNA downregulation of class II transactivator (CIITA), the central transcriptional activator of MHC class II genes. By repressing CIITA, HCMV-encoded factors prevent IFN-γ from inducing HLA-DR expression [111,112]. In addition, MHC class I downregulation is primarily mediated by several HCMV immune evasion proteins encoded within the US region. US2 and US11 promote the dislocation of newly synthesized MHC class I molecules from the endoplasmic reticulum (ER) to the cytoplasm, where they are subsequently degraded by proteasomes [113,114]. US6 inhibits transporter associated with antigen processing (TAP)-mediated peptide transport into the ER by binding directly to the TAP heterodimer, thereby preventing MHC class I molecules from acquiring antigenic peptides [115]. In contrast, US3 inhibits MHC class I by directly complexing with newly assembled MHC class I molecules and retaining them within the ER. This delay in transportation from the ER to the Golgi apparatus subsequently reduces MHC class I surface presentation in infected cells [116].

Together, these CMV immune-evasion strategies limit peptide loading onto MHC molecules and reduce the amount of peptide-MHC complexes displayed for T cell recognition [117]. As a result, the activation threshold for CD8+ T cells increases, favoring immune evasion. When infected cells display fewer viral peptide-MHC complexes, low-avidity CD8 T cells may no longer detect or respond to them, whereas high-avidity CD8+ T cells can still respond [118]. Together, these mechanisms can blunt antigen presentation to both CD8+ and CD4+ T cells [64,117].

CMV infection also suppresses host cell death pathways to ensure the survival of the infected cells. The viral inhibitor of caspase-8 (vICA), encoded by UL36, targets TNF death receptor signaling, a key mediator of extrinsic apoptosis. By preventing caspase-8 activation, vICA protects infected cells from CD8+ T-cell-mediated killing [119]. Additionally, intrinsic apoptosis is blocked through inhibitors such as vMIA (viral mitochondrial inhibitor of apoptosis) and vIBO (viral inhibitor of BAK oligomerization), which counteract the pro-apoptotic factors BAK and BAX, thereby preventing mitochondrial outer membrane permeabilization [119]. The viral protein UL38 has also been shown to suppress intrinsic apoptosis by inhibiting caspase-3 activation [120].

During CMV infection, immune-mediated apoptosis is regulated through the UL141 protein, which binds TRAIL death receptors with an affinity comparable to that of the natural TRAIL ligand, thereby limiting NK cell-mediated cytotoxicity [121].

Alternative programmed death pathways, such as necroptosis, are also counteracted through multiple strategies. For example, UL-36-encoded vICA has been reported to inhibit necroptosis by targeting MLKL (Mixed Lineage Kinase Domain-Like) and blocking receptor-interacting protein serine/threonine kinase 3 (RIPK3)-mediated cell death pathways [122,123].

In summary, these virus-encoded immune evasion strategies counteract host immune surveillance, prevent the elimination of infected cells, and facilitate viral persistence.

## 4. Drivers of CMV Reactivation in Kidney Transplantation

### 4.1. Host Immunosurveillance Disruption

**Figure 1:**
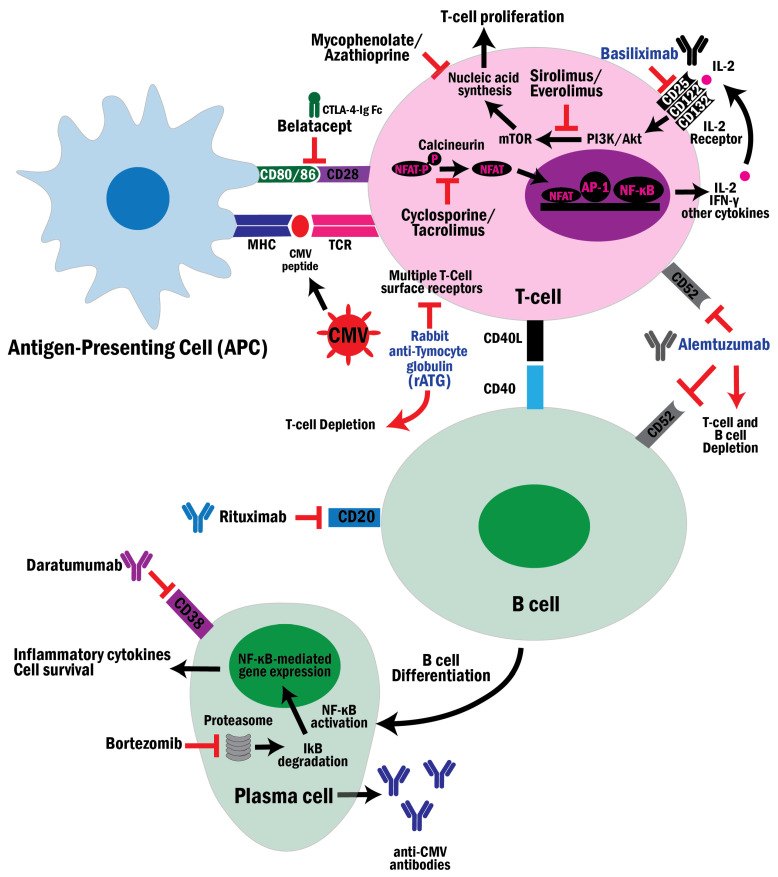
Overview of selected immune mechanisms involved in CMV infection control and how immunosuppressive therapies modulate them. CMV-specific CD4^+^ and CD8^+^ T cells provide primary antiviral control, supported by B cells (antibody production) and NK cells (innate immunity). Immunosuppressive agents impair these responses at multiple levels: T cell-depleting therapies reduce antiviral lymphocyte populations; calcineurin inhibitors and belatacept inhibit T cell activation; antimetabolites limit lymphocyte proliferation; and corticosteroids broadly suppress immune function. B-cell- and plasma-cell-targeted therapies reduce humoral immunity, while complement blockers impair innate responses. mTOR inhibitors have partial antiviral and immunomodulatory effects. Induction drugs shown in blue.

Immunosuppressive therapy influences innate and adaptive immunity, inflammatory signaling pathways, and host cellular mechanisms that regulate viral gene expression. Understanding how individual immunosuppressive drug classes modulate CMV-specific immune responses is critical for balancing rejection prevention and the risk of CMV infection in KTRs. Here, we discuss induction therapies, maintenance immunosuppression, B-cell- and plasma-cell-targeted therapies, and other rejection drugs currently used in clinical practice. Figure 1 illustrates the key targets of these immunosuppressive strategies and their effects on immune mechanisms involved in CMV infection control. Table 1 summarizes the CMV risks associated with immunosuppressive drug classes, their mechanism of action, and key clinical insights.

#### 4.1.1. Induction Drugs

The severity and duration of lymphocyte depletion, as well as the pattern of immune reconstitution, are critical determinants of the risk of CMV infection and reactivation. Induction agents differ substantially in their immunologic effects, translating into clinically meaningful differences in CMV infection and disease rates.

##### Rabbit Anti-Thymocyte Globulin (rATG)

Rabbit anti-thymocyte globulin (rATG; thymoglobulin) is a polyclonal antibody targeting multiple T-cell surface antigens. It induces rapid and profound T-cell depletion through complement-mediated cytotoxicity, apoptosis, and antibody-dependent cellular cytotoxicity. rATG also alters adhesion molecules, impairs dendritic cell function, and promotes regulatory T-cell expansion [124,125].

rATG depletes both naive and memory T-cell subsets, including CMV-specific CD4+ and CD8+ populations, which are essential for antiviral control. CD4+ T-cell recovery may remain delayed for months, resulting in impaired IFN-γ production and weakened suppression of the CMV IE gene expression [124,126]. The magnitude of the risk of CMV infection and reactivation correlates with the severity of lymphopenia and delayed immune reconstitution.

Multiple studies demonstrate increased CMV infection rates following rATG induction, especially in D^+^/R^−^ KTRs, compared with non-depleting regimens. Lymphocyte-depleting therapy also predisposes patients to late-onset CMV disease after completion of antiviral prophylaxis [127]. Similar associations between thymoglobulin and CMV infection have been observed in other solid organ transplant populations [128,129].

Importantly, in addition to lymphocyte depletion, rATG can induce cytokine release, which leads to increased levels of pro-inflammatory cytokines such as IL-6 [130,131,132] that promote IE gene expression and facilitate CMV reactivation [133].

##### Alemtuzumab

Alemtuzumab is a humanized monoclonal antibody targeting CD52, a molecule expressed on T and B lymphocytes. It induces profound and sustained lymphocyte depletion via complement-mediated lysis and antibody-dependent cytotoxicity [134,135]. CD4+ T cells recover particularly slowly, often requiring extended periods for reconstitution.

Depletion of CMV-specific memory T cells and reduction in antiviral cytokine production (IFN-γ, IL-2) significantly impair viral control [134]. Although NK cells may recover earlier, adaptive immune suppression persists.

Clinical studies have shown increased CMV DNAemia and reactivation rates following alemtuzumab induction compared with non-depleting strategies [134,135]. Impaired CMV-specific T-cell immunity has been demonstrated in recipients treated with alemtuzumab [136]. Both early reactivation and late-onset disease have been described, reflecting the depth and duration of immune suppression.

As with rATG, CMV infection in this context may contribute to inflammatory activation, rejection risk, and graft dysfunction.

##### Basiliximab

Basiliximab is a chimeric monoclonal antibody that targets the IL-2 receptor α-chain (CD25) on activated T cells, blocking IL-2–mediated clonal expansion without eliminating resting or memory lymphocytes, while preserving NK cell function, antigen-presenting cell activity, and anti-CMV immunosurveillance [137,138]. Comparative studies indicate a lower incidence of CMV infection with basiliximab than with rATG, particularly among D^+^/R^−^ recipients [139,140]. Outcomes remain favorable when combined with valganciclovir prophylaxis or preemptive strategies [141]. Basiliximab demonstrates comparable rejection outcomes with reduced infectious complications compared with low-dose rATG in low-risk living donor transplantation [140].

Overall, basiliximab offers effective rejection prophylaxis in low-to-moderate-risk recipients while minimizing CMV-related complications.

#### 4.1.2. Maintenance Immunosuppression

##### Calcineurin Inhibitors

Calcineurin inhibitors (CNIs) are the cornerstone of maintenance immunosuppression in KTRs. Both cyclosporine and tacrolimus suppress T cell activation by blocking calcineurin-dependent transcription of IL-2 and other cytokines. Cyclosporine binds cyclophilins, while tacrolimus binds FK-binding proteins [142,143]. These drug–protein complexes inhibit calcineurin, preventing the nuclear translocation of the nuclear factor of activated T cells (NFAT), thereby reducing transcription of IL-2, IL-3, IL-4, granulocyte-macrophage colony-stimulating factor, IFN-γ, and TNF-α, and leading to reduced T lymphocyte proliferation. NFAT-regulated cytokines normally create an antiviral environment. Their suppression results in loss of immune surveillance. Under calcineurin inhibition, CMV-specific effector T cell expansion and cytotoxic clearance of infected cells are impaired, favoring viral reactivation [144,145,146]. Several observational studies have shown that CNI-based immunosuppression is associated with high rates of CMV viremia and disease, particularly at higher drug levels. Some studies suggest that cyclosporine, rather than tacrolimus, and a higher overall burden of immunosuppression are associated with the development of CMV and other viremias [147,148]. However, another study found no difference in CMV infection risk between tacrolimus and cyclosporine. Tacrolimus exposure has also been associated with higher rates of CMV infection within 3 months post-transplant.

Importantly, the intensity of immunosuppression appears to be a stronger determinant of CMV infection risk than the type of CNI used, as regimens minimizing CNI exposure, such as those used in association with mTOR inhibitors, are associated with lower CMV incidence [149,150,151]. These clinical findings are supported by immunologic studies demonstrating a dose-dependent suppression of CMV-specific CD4+ and CD8+ T cell responses while on CNI therapies.

##### Antimetabolites

Antimetabolites are a major component of maintenance immunosuppression in kidney transplantation. Mycophenolate is the antimetabolite most widely used in KTRs, whereas azathioprine is now rarely used and is generally reserved for patients with contraindications to mycophenolate or those who develop significant mycophenolate-related adverse effects [152,153]. They inhibit both T- and B-lymphocyte proliferation by blocking nucleic acid synthesis, thereby weakening the adaptive immune surveillance that maintains CMV latency. Unlike CNIs, antimetabolites do not affect cytokine expression; instead, they limit immune cell expansion and humoral response.

The risk of CMV viremia did not differ significantly between mycophenolate- and azathioprine-treated patients; however, the risk of tissue-invasive CMV disease was higher in mycophenolate-treated patients than in azathioprine-treated patients [152,153,154].

##### mTOR Inhibitors

mTOR-inhibitor-based immunosuppression is consistently associated with a lower incidence of CMV infection compared with CNI-based regimens. At the mechanistic level, mTOR inhibitors (sirolimus, everolimus) suppress mTORC1-dependent protein translation by inhibiting phosphorylation of downstream effectors involved in cap-dependent mRNA translation and attenuate inflammatory signaling pathways that contribute to NF-κB activation. This results in reduced protein synthesis and may also decrease cytokine-mediated activation of the CMV MIEP [155].

Clinically, meta-analyses demonstrated a significant reduction in CMV events among patients receiving mTOR inhibitors, as well as lower rates of CMV reactivation and recurrence.

Conversion from CNI to sirolimus in selected KTRs has been associated with reduced CMV episodes, suggesting improved control of viral replication and possibly enhanced antiviral immune surveillance under mTOR-based therapy [156]. Additionally, mTOR inhibitors increase the frequency and functional capacity of CMV-specific cytotoxic γδ T cells and αβ T cells involved in the antiviral response, potentially improving antiviral immune surveillance [157].

These findings have implications for prophylaxis strategies. Given the reduced CMV burden observed with mTOR inhibitors, the necessity and duration of universal antiviral prophylaxis may be reconsidered in certain low-to-moderate-risk populations, although decisions remain individualized and risk-dependent [155].

##### Corticosteroids

Corticosteroids remain fundamental to immunosuppressive regimens after kidney transplantation but increase the risk of reactivation of latent viral infections, particularly CMV. Corticosteroids suppress T-cell proliferation, reduce cytokine production (IL-2, IFN-γ), and impair antigen presentation, thereby weakening CMV-specific immune surveillance. High-dose or pulse steroid therapy, often used to treat rejection episodes, markedly increases the risk of CMV reactivation and subsequent viral replication, especially in high-risk donor–recipient serostatus pairs (D^+^/R^−^) [158,159]. They can activate the mineralocorticoid receptor (MR), whose transcriptional response varies according to genetic polymorphisms such as rs5522 (Ile180Val). The Val-180 variant alters receptor sensitivity to cortisol, potentially modifying the biological “effective dose” of corticosteroids and contributing to inter-individual variability in immunosuppressive depth [160].

#### 4.1.3. B Cell and Plasma Cell Targeted Therapies

B-cell and plasma-cell-directed therapies are used in KTRs for desensitization, antibody-mediated rejection, and treatment of recurrent glomerular disease.

Rituximab is a monoclonal antibody against CD20 that depletes circulating B cells but does not affect plasma cells. It has been used in kidney transplantation for desensitization in highly sensitized recipients and in ABO incompatible kidney transplants, treatment of antibody-mediated rejection (ABMR), recurrence of some glomerular disease, and in post-transplant lymphoproliferative disorder (PTLD).

B cells are antigen-presenting cells and cytokine producers; thus, B cell blockade alters T cell priming, increasing the risk of infections, including viral infections [161]. Clinical findings on CMV risk with rituximab are heterogeneous. Some studies reported no increased risk of CMV infection with rituximab use [162,163,164]. Although some studies did not show a difference in CMV infection in KTRs who received rituximab compared with KTRs who received standard therapy, a tendency towards higher CMV disease in the rituximab-treated recipients group has been observed [165,166]. Other studies reported increased CMV infection in KTRs treated with a single dose of rituximab in desensitization [167]. Notably, increased incidence of CMV has been reported when rituximab is used in combination with other therapies like rATG or intravenous immunoglobulin (IVIG).

Bortezomib (a proteasome inhibitor) and daratumumab (a monoclonal antibody targeting CD38) are increasingly used in kidney transplantation to treat ABMR and to desensitize highly sensitized KTRs. By targeting plasma cells and modulating T-cell and NK cell functions, these agents may increase susceptibility to infections, including CMV. However, their effects in kidney transplantation are not well defined, as studies are limited and data are heterogeneous [168,169,170].

#### 4.1.4. Other Anti-Rejection Drugs

##### Interleukin-6 Inhibitors

IL-6 is a key cytokine involved in B-cell differentiation, maintenance of T follicular helper (Tfh) cells, effector T-cell expansion, and acute-phase inflammatory responses. IL-6 inhibitors such as tocilizumab (an IL-6 receptor blocker) and clazakizumab (an anti-IL-6 ligand) are increasingly used for chronic active ABMR. Their therapeutic effects include reducing donor-specific antibody (DSA) production by suppressing plasma cell differentiation, inhibiting germinal center activity, and attenuating endothelial inflammatory injury.

IL-6 signaling induces CMV reactivation and supports CMV-specific CD4+ and CD8+ T-cell expansion and function, which are critical for maintaining CMV latency [171]. Blockade of IL-6 may therefore impair antiviral T-cell proliferation and polyfunctionality, reduce CD4+ T-cell help, and blunt inflammatory signaling that restrains viral replication. IL-6 inhibition may destabilize immune control of latent CMV when added to the baseline immunosuppression [158,172,173,174].

Although IL-6 inhibitors improve graft outcomes in chronic ABMR, their immunomodulatory effects carry infection-related risks, including increased CMV viremia risk, particularly in CMV-seropositive or heavily immunosuppressed recipients, delayed recognition of infection due to suppression of IL-6–mediated acute-phase markers such as CRP, and potentially prolonged viral replication, reflecting impaired cellular immune surveillance [175,176].

#### 4.1.5. Belatacept and Disruption of Antiviral Immunosurveillance in CMV Latency and Reactivation

Belatacept-based immunosuppression has been increasingly associated with higher rates of CMV infection and reactivation in transplant recipients, underscoring the need to understand how co-stimulatory blockade influences antiviral immune surveillance. Belatacept is a selective co-stimulation blocker that binds CD80/CD86 on antigen-presenting cells and prevents engagement with CD28 on T lymphocytes, thereby inhibiting the second signal required for effective T-cell receptor (TCR)-mediated activation, proliferation, and IL-2 production [177]. Structurally, belatacept is a fusion protein composed of the extracellular domain of CTLA-4 (Cytotoxic T lymphocyte-associated protein 4) linked to a modified human IgG1 Fc fragment with high affinity for CD80/CD86, allowing suppression of alloimmune responses while avoiding the nephrotoxicity associated with CNIs.

Although belatacept prevents allograft rejection by blocking CD80/CD86–CD28 costimulatory signaling, this pathway is also relevant for the generation and maintenance of effective T-cell responses. CMV persists lifelong in a latent state, but latency is dynamic and may be interrupted by episodes of viral gene expression or reactivation, particularly under immunosuppression [1,178]. In this setting, CMV-specific CD4^+^ and CD8^+^ T cells provide immune surveillance that limits viral replication upon reactivation [93,179]. Therefore, by interfering with CD28-dependent costimulation, belatacept may compromise the development of functional quality of CMV-specific T-cell immunity in susceptible patients, reducing the speed or efficacy of immune control after CMV reactivation and thereby increasing the risk of clinically significant CMV replication or disease [180,181].

The impact of costimulatory blockade on CMV immunity depends, in part, on T-cell maturation. CD28 signaling is essential for the activation and expansion of naïve T cells, but is less critical for differentiated memory T cells [182]. Experimental studies support this distinction. In an in vitro study using peripheral blood mononuclear cells from CMV-seropositive and CMV-seronegative donors, stimulated with CMV pp65 peptides, belatacept inhibited activation of naïve T cells, with a lesser effect on differentiated CMV-specific T cells. These differentiated cells retained the ability to produce antiviral cytokines, including TNF-α, IL-2, and IFN-γ, indicating that CMV-specific memory responses are relatively less dependent on CD28-mediated co-stimulation [183].

This distinction has important clinical implications in transplantation. In CMV-seronegative recipients receiving organs from CMV-seropositive donors (D^+^/R^−^), protective immunity must be generated de novo from naïve T-cell precursors. Because CD28-mediated co-stimulation is required for naïve T-cell priming and clonal expansion, belatacept blockade may impair the development of effective CMV-specific immunity in these high-risk recipients. Consistent with this mechanism, belatacept-based immunosuppression has been associated with higher rates of CMV viremia, prolonged viral replication, and delayed viral clearance compared with CNI-based immunosuppression regimens [184].

Chronic CMV infection promotes memory inflation and T-cell exhaustion, creating an impaired immune environment that may be further exacerbated by costimulatory blockades [181,185]. Recent data suggest that, in addition to blocking CD28-mediated costimulation, belatacept may influence CMV control through “checkpoint” pathways [181]. In particular, PD-L1 can interact with CD80 on antigen-presenting cells, thereby modulating PD-1-mediated inhibitory signaling. Because belatacept binds to CD80 and CD86, it has been hypothesized that it might alter this balance and contribute to dysfunctional or exhausted phenotypes of CMV-specific T-cell responses in some transplant recipients [186,187].

When belatacept binds CD80, these CD80–PD-L1 complexes may be disrupted, increasing the availability of free PD-L1 that can interact with PD-1 receptors on T cells and suppress their activity [181]. Enhanced PD-1 signaling may promote progressive T-cell exhaustion during chronic viral antigen exposure. Furthermore, conversion from tacrolimus-based immunosuppression to belatacept may remove inhibition of the NFAT–TOX transcriptional pathway, a molecular axis implicated in irreversible T-cell exhaustion [181].

CMV itself further contributes to immune dysregulation through viral immune evasion mechanisms, including interference with antigen presentation and induction of inhibitory checkpoint ligands, such as PD-L1, on infected cells. Together, these mechanisms suggest that susceptibility to CMV infection during belatacept therapy results from the combined effects of impaired naïve T-cell priming, chronic CMV-driven memory inflation, accelerated immunosenescence, checkpoint-mediated T-cell exhaustion, and viral immune-evasion strategies that collectively weaken antiviral immune surveillance [181].

Clinically, CMV reactivation in belatacept-treated recipients has been associated with higher viral loads, recurrent CMV episodes, and increased risk of tissue-invasive disease. Indirect effects may include increased susceptibility to opportunistic infections, heightened risk of acute rejection, and potential allograft dysfunction. These observations highlight the importance of individualized risk assessment, intensified CMV monitoring, and tailored immunosuppressive strategies when belatacept is used, particularly in high-risk CMV serostatus combinations.

**Table 1:** CMV risks across immunosuppressive drug classes in kidney transplantation. High: consistently associated with high rates of CMV infection in clinical trials or registry data, often requiring universal antiviral prophylaxis. Moderate: associated with increased risk in a specific combination (with T-cell-depleting agents) or in a high-risk population. Low: minimal impact on CMV-specific memory T cell or viral replication, generally considered safe. Reduced: unique to mTOR inhibitors; these agents demonstrate an intrinsic antiviral effect that can lower the baseline risk of infection. Abbreviations: rATG: Rabbits Anti-Thymocyte Globulin, CNIs: calcineurin inhibitors, mTOR Inhibitors: Mammalian Target of Rapamycin Inhibitors, D^+^/R^−^: CMV-seropositive donor and CMV-seronegative recipient (highest risk pair), NF-κB/NFAT: Intracellular signaling pathways/transcription factors critical for immune activation and viral gene expression, NK cells: Natural killer cells, Tfh: T-follicular helper cells.

Drugs	Mechanism	CMV Risk	Key Clinical Insight	References
rATG	Profound T cell depletion (naive and memory); delays immune reconstitution.	High CMV risk	Significantly higher incidence in D^+^/R^−^ pairs; risk of late-onset disease; induces expression of inflammatory cytokines.	[124,125,126,127,128,129,130,131]
Alemtuzumab	Sustained depletion of CD52+ cells (T and B cells); slow CD4+ recovery.	High CMV risk	Increased CMV viremia and reactivation compared to non-depleting agents; induces expression of inflammatory cytokines.	[134,135,136]
Basiliximab	Blocks IL-2 receptor on activated T cell; preserves memory T cell pool.	Low CMV risk	Lower CMV incidence compared to rATG; maintains immunosurveillance.	[137,138,139,140,141]
Calcineurin inhibitors (CNIs)	Block NFAT-dependent cytokine transcription (IL-2, IFN γ); impair T cell expansion.	High CMV risk	Dose-dependent risk; intensity of overall immunosuppression is the key driver.	[142,143,144,145,146,147,148,149,150,151]
Antimetabolites	Inhibit T and B cell proliferation, weaken adaptive surveillance.	High CMV risk	Higher risk of tissue-invasive disease in mycophenolate compared to azathioprine.	[152,153,154]
mTOR Inhibitors	Inhibit viral protein translation and dampens inflammatory (NF-κB) signaling.	Reduced CMV risk	Associated with significantly lower CMV incidence and reduced recurrence.	[155,156,157]
Corticosteroids	Suppress T cell proliferation and cytokine production; impair antigen presentation.	Variable and dose-dependent CMV risk	Pulse therapy markedly increases risk of CMV.	[158,159,160]
Belatacept	Selective T cell co-stimulation blockade (CD80/86-CD28).	High CMV risk	Linked to higher incidence of atypical and refractory viremia.	[177,182,183,184,185]
Rituximab	Depletes B cells; alters T cell priming, cytokine production, and cyclical production.	Moderate CMV risk	Conflicting studies show a tendency toward higher CMV disease rates; induces expression of inflammatory cytokines.	[161,162,163,164,165,166,167]
Bortezomib	Proteasome inhibition; induces plasma cell apoptosis and impairs dendritic cells and T cell response.	Moderate CMV risk	Risk in multiple myeloma; data in kidney transplant is limited, but suggests risk.	[168]
Daratumumab	Depletes CD38+ plasma cells, NK cells, and activated T cells.	Moderate CMV risk	Increased viral risk noted in multiple myeloma; larger transplant studies are still needed.	[169,170]
IL-6 Inhibitors	Impairs Tfh maintenance and T cell expansion I, blunts inflammatory signals.	Unclear CMV risk	May attenuate inflammatory triggers of CMV reactivation; suppresses IL-6-mediated CRP and potentially delays diagnosis.	[158,172,173,174,175,176]

### 4.2. Tissue Injury and Inflammation as Drivers of CMV Reactivation in Kidney Transplantation

**Figure 2:**
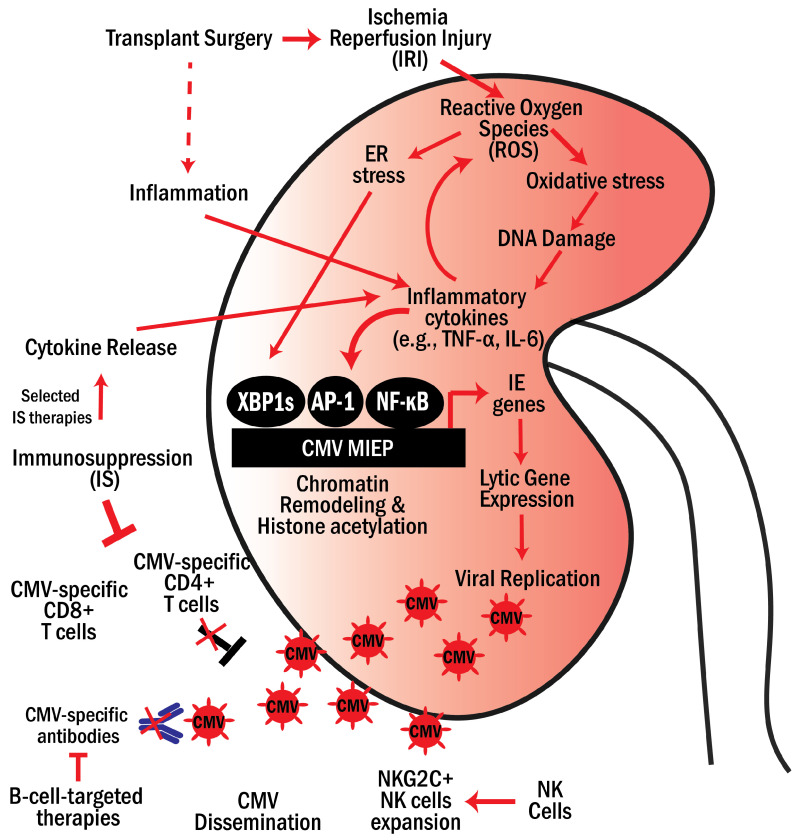
Key mechanisms driving CMV reactivation following kidney transplantation. Transplant surgery triggers ischemia–reperfusion injury (IRI), leading to the production of reactive oxygen species (ROS), oxidative stress, DNA damage, and endoplasmic reticulum (ER) stress. These processes result in the production of pro-inflammatory cytokines, including TNF-α and IL-6, which activate transcription factors such as AP-1 and NF-κB, while ER stress activates XBP1. These transcription factors promote epigenetic remodeling of the major immediate-early promoter (MIEP), leading to transcription of CMV immediate-early genes, and subsequent viral lytic gene expression, replication, and dissemination. Immunosuppressive therapies affect CD4+ and CD8+ T-cell responses, reducing immunosurveillance, while B-cell-targeted therapies impair humoral immunity, including the production of CMV-specific antibodies. In addition, selected immunosuppression regimens can induce cytokine release, further amplifying cytokine-driven viral reactivation. In kidney transplantation, CMV reactivation is also linked to the expansion of NKG2C+ NK cells.

CMV reactivation in kidney transplantation is driven by the interplay between tissue injury, inflammatory signaling, and immunosuppressive therapies. IRI and perioperative immune alterations generate inflammatory signals that create a permissive environment for viral reactivation. In parallel, immunosuppressive regimens impair the host antiviral immune surveillance, enabling uncontrolled viral replication and dissemination and, in some cases, induce the release of pro-inflammatory cytokines. The convergence of drug-induced and injury-induced cytokines, including TNF-α and IL-6, promotes the activation of lytic viral gene expression and viral replication. These mechanisms are summarized in Figure 2.

#### 4.2.1. Tissue Injury, Oxidative Stress, and Damage-Associated Signals

Growing evidence suggests that host tissue injury and cellular stress can contribute to the reactivation of latent CMV. During allogeneic transplantation, the blood supply to the donor organ is temporarily interrupted before being restored after the organ is grafted into the recipient. This ischemia–reperfusion process produces significant cellular stress, particularly within mitochondria. Oxidative damage following restoration of blood flow during reperfusion leads to endothelial cell dysfunction, DNA damage, and the release of damage-associated molecular patterns (DAMPs). DAMPs are recognized by PRRs on innate immune cells, triggering downstream signaling that activates innate immune responses and promotes neutrophil infiltration, which further amplifies oxidative stress within the injured tissue [188]. For example, PRRs such as Toll-like receptors (TLR-2 and TLR4) can respond to several DAMPs, including High Mobility Group Box 1 (HMGB1), and activate NF-κB and AP-1 via MAPK-IKK-MyD88 signaling, leading to cytokine production [189,190]. NF-κB and AP-1 bind to regulatory elements within the CMV MIEP, driving transcriptional activation of viral IE genes.

Transplant-associated IRI induces ER stress and mitochondrial dysfunction through TLR and Myd88 [191,192,193]. Inositol-requiring enzyme-1α (IRE-1α), the principal ER stress transducer, is activated in both IRI and viral infection, leading to mRNA splicing and the expression of the transcription factor X-box binding protein 1 (XBP1), and promoting inflammatory signaling and metabolic adaptation that can facilitate CMV replication [194]. IRE-1α mediates unconventional splicing of XBP1 to generate the active transcription factor XBP1s [195,196]. IRE-1α/XBP1 activation is also induced early in both MCMV and HCMV infection [197,198]. XBP1s directly bind the MCMV MIEP, promoting IE gene expression [198,199]. Conversely, CMV induces ER stress early in infection and modulates IRE-1α/XBP1 activity to optimize replication while avoiding excessive pro-apoptotic signaling. In parallel, the mitochondrial fission regulator dynamin-related protein 1 (DRP1) is frequently activated during viral infection, leading to mitochondrial fragmentation, increased reactive oxygen species (ROS), augmented ER stress, metabolic reprogramming, and altered innate immune signaling [200,201].

Hypoxia during ischemia disrupts the mitochondrial electron transport chain, leading to ROS accumulation and the activation of transcription factors such as NF-κB and AP-1 [202]. In this way, host tissue injury and cellular stress can directly stimulate viral reactivation [28]. In murine transplantation models, pathways associated with oxidative stress and DNA damage were upregulated in donor kidneys following IRI, regardless of whether the recipients were immunosuppressed. These findings suggest that inflammatory signaling triggered by tissue injury alone can promote CMV reactivation independently of immune suppression [63,76,79,82,203]. In summary, tissue injury and inflammation induced during transplant contribute to creating a permissive environment that favors CMV reactivation.

Additionally, because endothelial cells in the graft can serve as sites of latent CMV infection, viral reactivation in these cells may contribute to disease pathogenesis through viral replication, induction of inflammatory cytokines, endothelial activation, and promotion of vascular injury. These processes have been implicated in CMV-associated complications, including tissue-invasive disease and potential interactions with allograft injury; however, the direct contribution of endothelial CMV infection to chronic graft dysfunction remains incompletely defined [204,205].

#### 4.2.2. Inflammatory Signaling and Cytokine-Mediated Reactivation

CMV latency exists as a delicate equilibrium that can be disrupted by pro-inflammatory cytokines, leading to viral reactivation. Pro-inflammatory cytokines, including TNF-α and IL-1β, can activate pathways that promote viral transcription in latently infected cells. Elevated IL-6 and related mediators can drive the differentiation of monocytes and progenitor cells harboring latent virus, creating a cellular state permissive for transcription and replication. This link between cellular activation and reactivation has been documented in both experimental and clinical studies [133]. In murine models, TNF-α has been shown to trigger IE transcription and MIEP activation, concomitant with NF-κB activation. In both HCMV and MCMV, IE1 and IE3 signal the initiation of a pro-inflammatory cascade that, in turn, disrupts latency [76,206]. However, in murine kidney transplant models, TNF-α has been shown not to be required for CMV reactivation, and TNF-independent pathways can initiate latent CMV reactivation [207]. Notably, human post-renal transplant patients with CMV reactivation had higher levels of soluble interleukin-2 receptor (IL-2R), IL-6, and IL-10, and lower levels of IFN-γ. Elevated levels of IL-10 may suppress IFN-γ production, thereby reducing Th1-mediated antiviral activity and promoting reactivation.

In addition to injury-derived inflammation, immunosuppressive therapies can also promote cytokine production, further contributing to CMV reactivation. As discussed in Section 4.1, lymphocyte-depleting agents such as rATG and alemtuzumab are known to induce cytokine release. rATG administration is associated with infusional-related cytokine release and increased levels of several pro-inflammatory cytokines, including TNF-α and IL-6, as observed in both allogeneic hematopoietic stem cell transplantation and kidney transplantation [130,131,132]. Similarly, alemtuzumab induces cytokine release, with increased levels of TNF-α, IL-6, and IFN-γ in human studies, a finding also supported in humanized CD52-transgenic mice following CD52 depletion [208]. Rituximab, which targets CD20 on B cells, without impairing T-cell responses, also induces cytokine release (e.g., IL-6), but to a lesser extent and transitionally compared to anti-T-cell agents [209].

These cytokines are known to activate signaling, such as NFκB and AP1, that regulate the CMV MIEP and drive lytic gene expression. Notably, basiliximab, an anti-IL2 receptor monoclonal antibody, is not associated with cytokine release and has been shown to be associated with a lower incidence of CMV reactivation compared with depleting agents [210]. Together, these observations suggest that immunosuppression agents may promote reactivation not only by impairing antiviral immune surveillance but also by contributing to the pro-inflammatory environment that favors viral reactivation.

## 5. Clinical Challenges and Future Directions

### 5.1. Limitations of Current Antiviral Strategies

CMV management in kidney transplantation aims to prevent viral reactivation, particularly in high-risk D^+^/R^−^ recipients, using universal prophylaxis and preemptive therapy. Universal prophylaxis involves administering antivirals (e.g., valganciclovir and ganciclovir) for a defined period post-transplant to suppress viral replication, whereas preemptive therapy relies on regular viral load monitoring to initiate treatment when replication is detected [211,212]. While both strategies reduce early CMV disease, important limitations persist. Current surveillance relies primarily on plasma CMV PCR, as urine CMV PCR may detect viral shedding but has limited specificity for clinically significant infection or prediction of CMV disease. Plasma CMV DNAemia better reflects systemic replication and guides therapy, while the role of compartment-specific CMV monitoring within the kidney allograft remains investigational [213].

Universal prophylaxis may lead to late-onset CMV infection after drug cessation, suggesting delayed immune reconstitution rather than eradication of viral latency [173,214].

Other limitations of antivirals, such as valganciclovir, include hematologic toxicity, especially leukopenia and neutropenia [215], and the development of antiviral resistance via UL97 and UL54 mutations, particularly in high-risk or heavily immunosuppressed patients [216].

These limitations also apply to newer antiviral agents, including letermovir and maribavir. Letermovir offers reduced myelotoxicity but has limited data in kidney transplantation and documented resistance pathways [217]. Maribavir improves outcomes in refractory CMV infection but is less effective in tissue-invasive disease and may select resistance mutations [218].

Importantly, prolonged pharmacologic suppression may impair the development of CMV-specific T-cell immunity, predisposing patients to reactivation once therapy is discontinued [174].

Preemptive strategies reduce drug exposure but require intensive viral load monitoring and may miss early viral replication, particularly in highly immunosuppressed hosts and high-risk D^+^/R^−^ patients, who may rapidly progress from low-level viremia to symptomatic disease in days. Moreover, low-level viremia may exert indirect immunomodulatory effects that contribute to graft dysfunction and alloimmune activation, and tissue-invasive disease (e.g., gastrointestinal involvement) may occur in the absence of detectable CMV viremia [219,220].

Importantly, preemptive therapy allows a period of viral replication before initiation of antiviral therapy, which may trigger alloimmune activation and endothelial dysfunction.

Critically, current antivirals suppress viral replication but do not eliminate latent reservoirs or fully prevent CMV-induced immune activation, endothelial dysfunction, and enhanced alloimmunity, key mechanisms associated with rejection and chronic graft injury [158].

Current antiviral prophylaxis and treatment strategies reduce early CMV disease after kidney transplantation but are limited by late-onset infection, drug toxicity, resistance, and incomplete control of CMV-mediated immunomodulation. Future approaches should integrate immune monitoring and novel immunotherapeutic strategies to address the persistent clinical and immunological impact of CMV reactivation. These include: (1) Immune-monitoring-guided prophylaxis using CMV-specific T-cell assays that may personalize duration and reduce late disease, (2) Adoptive CMV-specific T-cell therapy and vaccine strategies, which are under investigation but require validation in solid organ transplantation, and (3) Integrated approaches targeting both viral replication and CMV-driven immune dysregulation may be necessary to improve long-term graft outcomes [221].

### 5.2. CMV Monitoring, Immunomonitoring, and Risk Stratification

The prevailing standard for CMV surveillance in kidney transplantation relies on quantitative nucleic acid amplification testing (qNAT) of plasma or whole blood [173]. However, qNAT primarily detects systemic viral replication and may not capture the early stages of viral reactivation. Consequently, intervention thresholds are often reached after the virus has overcome initial immune containment, potentially resulting in delayed treatment and cumulative drug toxicity from prolonged prophylaxis regimens.

A considerable limitation of qNAT is a lack of universal standardization. Viral load thresholds for initiating therapy vary widely across centers, with no consensus on clinically actionable cutoffs [222]. In addition, qNAT does not provide information about the functional capacity of the host immune system to control CMV infection, which is arguably the most clinically relevant determinant of reactivation risk [223].

To address these limitations, CMV-specific cell-mediated immunity (CMI) assays, such as ELISpot and IFN-release assays, have been developed to primarily assess CMV-specific T-cell responses. Across multiple observational and interventional studies, low or absent CMV-specific T-cell responses at defined post-transplant timepoints are consistently associated with subsequent viral replication, whereas preserved responses predict spontaneous clearance and reduced antiviral requirements [224,225,226].

However, these assays face similar limitations to qNAT in clinical adoption, including poorly defined, unstandardized positivity thresholds across platforms, variability in antigen stimulation protocols, and the absence of head-to-head comparisons between assays. Immunosuppression itself, particularly induction therapy with rATG, can significantly suppress CMV-specific T cell responses and alter the predictive accuracy of these assays depending on the timepoint at which they are performed post-transplant.

Traditional risk stratification based on donor and recipient serostatus is central in clinical practice. However, serostatus alone is proving to be insufficient, as it does not account for variability in immune competence among SOT recipients [227]. Evidence shows that CMV-specific CD8+ T cells can protect CMV-seronegative recipients; by contrast, a low T cell response post-transplant is associated with an increased risk of reactivation [228]. This variability is undetected by standard serologic testing and can therefore lead to under- or over-treatment. Post-transplant immune recovery is influenced by individual patient factors, and single-timepoint immune measurements may fail to capture transient vulnerabilities, underscoring the need for longitudinal monitoring of CMV-specific T cell function [229].

Combining CMV-specific T cell assays with traditional serological viral load measurements may allow for more precise risk assessment [230]. This approach, in conjunction with longitudinal immunomonitoring, could be used to tailor interventions such as the timing and duration of antiviral prophylaxis [231]. Integration of virologic and immunologic monitoring provides a more comprehensive assessment of CMV reactivation risk and may enable more personalized management of CMV infection in KTRs.

### 5.3. Emerging Strategies for Improving CMV Infection Control in Kidney Transplantation

Several emerging strategies are currently under evaluation to improve CMV infection control, including monoclonal antibodies targeting CMV antigens, T-cell-based therapies, vaccination strategies, and epigenetic approaches.

Among monoclonal antibodies against CMV antigens, Fiztasovimab (NPC-21), which targets the viral glycoprotein B, was recently evaluated in a phase 2 clinical study in D^+^/R^−^ KTRs. Although safe, the treatment did not significantly reduce CMV infection at 16 weeks. Further studies are needed to determine its potential role in preventing severe CMV disease [232].

T-cell-based strategies, including the adoptive transfer of CMV-specific T cells, have been investigated in transplantation, primarily in hematopoietic stem cell transplantation (HSCT), with limited data in solid organ transplantation [233]. In one study, infusion of in vitro-expanded autologous CMV-specific T cells has been associated with improved clinical outcomes in SOT recipients with recurrent or ganciclovir-resistant CMV infection [234]. A phase 2 clinical study evaluating the infusion of third-party CMV-specific T cells in SOT recipients also showed clinical benefit, with a 65% response rate [235]. These findings are promising, but larger studies are needed to confirm the efficacy and feasibility of CMV-specific T cell therapy in KTRs.

CMV vaccines are also under examination in kidney transplantation. For example, the bivalent recombinant vaccine HB-101, which uses viral gB and pp65 to stimulate T-cell and antibody responses, was evaluated in CMV-seronegative kidney transplant recipients, but unfortunately, the administration of two doses did not show a significant decrease in CMV infection and disease compared to the placebo in a phase 2 clinical trial (NCT03629080). Similarly, the DNA-based vaccine ASP0113 was not effective in preventing CMV viremia in CMV-seronegative KTRs in another phase 2 study (NCT01974206) [236]. Although an mRNA-based vaccine, mRNA-1647, showed promising results in phase 1 and 2 clinical trials [237], a phase 3 study (NCT05085366) conducted in a cohort of CMV-seronegative women of childbearing age did not demonstrate efficacy in preventing CMV infection. Together, these findings highlight the challenges of achieving immunoprotection against CMV through vaccination.

Epigenetic modulation of CMV latency and reactivation is also being explored as a potential therapeutic strategy. Approaches such as “shock and kill”, which aim at inducing cells to reactivate from latency to enable infected cell clearance by the host immune system, and “block and lock”, which aims to repress viral gene expression, preventing transcriptional activation to maintain CMV in a latent phase, have been proposed based on studies on HIV [238]. Histone deacetylase inhibitors, such as sodium valproate, have been proposed as agents that can promote partial CMV reactivation, induce limited expression of IE genes, and potentially trigger the activation of a CMV-specific response. Bromodomain and Extra-Terminal domain (BET) inhibitors have also emerged as agents that can induce partial viral gene expression by inhibiting c, potentially enabling the elimination of infected cells [39]. Although promising, epigenetic therapies are preclinical and have several critical limitations, including limited specificity, off-target effects, and toxicity. In addition to strategies aimed at modulating epigenetic reprogramming of host immune cells, this could represent another area worth investigating.

Finally, tolerance-inducing strategies have also been investigated in kidney transplantation to reduce the need for long-term immunosuppression [239,240]. Approaches such as mixed chimerism and cellular therapies that enable withdrawal of immunosuppression may help control CMV infection by preserving antiviral immunity. However, their effect on CMV reactivation remains to be evaluated.

In summary, while several innovative strategies are under consideration, their clinical translation into effective treatments for CMV infection and disease remains a challenge.

## 6. Conclusions

CMV remains a major infectious complication following kidney transplantation, driven by the disruption of the balance between viral latency and host immune control. Collectively, CMV reactivation in KTRs could be explained as the convergence of three main processes: (1) epigenetic derepression of the viral genome, (2) inflammatory signaling that activates the MIEP, and (3) impaired immune surveillance due to immunosuppression.

Despite effective antiviral prophylaxis and treatment strategies, several limitations remain in CMV management in KTRs, including drug toxicity, late-onset infection, emergence of antiviral resistance, and the inability to target latently infected cells and prevent reactivation. The highest risk of CMV infection occurs in CMV-seronegative recipients receiving organs from CMV-seropositive donors (D^+^/R^−^), particularly during the early post-transplant period or after discontinuation of antiviral prophylaxis. Lymphocyte-depleting agents, such as rATG and alemtuzumab, that induce inflammatory cytokine expression pose the greatest risk by activating IE gene expression and impairing T cell immunity. In contrast, non-depleting agents such as basiliximab, which do not induce cytokine expression, preserve memory lymphocytes, thereby reducing CMV incidence. Overall, the type and intensity of immunosuppression appear to play an important role in CMV reactivation after kidney transplantation.

Future approaches, including targeted immunosuppression, immunomonitoring-guided therapy, adoptive T-cell strategies, and exploration of epigenetic interventions, may better preserve antiviral immunity while maintaining adequate protection against rejection.

In conclusion, integrating mechanistic insights into clinical management will enable a more precise, patient-centered approach to control CMV infection in kidney transplantation.

## Data Availability

No new data were created or analyzed in this study. Data sharing is not applicable to this article.

## Abbreviations

The following abbreviations are used in this manuscript:

ABMR	Antibody-mediated rejection
ADTELs	Antigenicity-determining transcripts expressed in latency
AP-1	Activator protein 1
ATRX	Chromatin remodeler ATRX
BAF	BRG1-associated factor chromatin remodeling complex
BET	Bromodomain and Extra-Terminal domain
BRD4	Bromodomain protein 4
CCL3	C-C Motif Chemokine Ligand 3
CCL4	C-C Motif Chemokine Ligand 4
CD	Cluster of differentiation
CMV	Cytomegalovirus
CNI	Calcineurin inhibitor
CREB	cAMP response element binding protein
CRP	C-reactive protein
CTCF	CCCTC binding protein
CTLA-4	Cytotoxic T lymphocyte-associated protein 4
CX3CR1	C-X3-C motif chemokine receptor 1
DAMPs	Damage-associated molecular patterns
DNMT1	DNA methyltransferase 1
DRP1	Dynamin-related protein 1
DSA	Donor-specific antibody
D^+^/R^−^	Donor seropositive/recipient seronegative
D^−^/R^−^	Donor seronegative/recipient seronegative
EGFR	Epidermal growth factor receptor
EGR-1	Early growth response 1
ER	Endoplasmic reticulum
ERK	Extracellular signal regulated kinase
EZH2	Enhancer of zeste homolog 2
Fc	Fragment crystallizable region
FSGS	Focal segmental glomerulosclerosis
GATA2	GATA binding factor 2
GR	Glucocorticoid responsive
HDAC	Histone deacetylase
HMGB1	High mobility group box 1
HP1	Heterochromatin protein 1
HPCs	hematopoietic progenitor cells
H3K9me3	Histone 3 trimethylated on lysine 9
H3K27me3	Histone 3 trimethylated on lysine 27
HCMV	Human cytomegalovirus
IE	Immediate early
IFN-γ	Interferon gamma
IL	Interleukin
IL-2R	Interleukin 2 receptor
IRE-1α	Inositol-requiring enzyme-1α
IRI	Ischemia–reperfusion injury
KAP1	KRAB-associated protein 1
KIRs	Killer cell immunoglobulin-like receptors
KTRs	Kidney transplant recipients
LUNA	Latency unique natural antigen
MAPK	Mitogen-activated protein kinase
MCMV	Murine cytomegalovirus
MHC	Major histocompatibility complex
MICB	MHC class I-related chain B
MIEP	Major immediate-early promoter
miRNAs	microRNAs
MR	Mineralocorticoid receptor
mTOR	Mammalian target of rapamycin
NFAT	Nuclear factor of activated T cell
NF-κB	Nuclear factor kappa B
NK cells	Natural killer cells
NKG2C	Natural killer group 2C
NKGD2	Natural killer group 2D
PAMPs	pathogen-associated molecular patterns
P-TEFb	Positive transcription elongation factor b
PD1	Programmed cell death protein 1
PD-L1	Programmed cell death ligand 1
PML-NBs	Promyelocytic leukemia nuclear bodies
PRC2	Polycomb repressive complex 2
PRRs	Pattern recognition receptors
qNAT	Quantitative nucleic acid amplification testing
RIPK3	Receptor-interacting protein serine/threonine kinase 3
ROS	Reactive oxygen species
rATG	Rabbit anti-thymocyte globulin
SEC	Super elongation complex
SETDB1	Histone-lysine N-methyltransferase
SFK	Src family kinase
SOT	Solid organ transplantation
TAP	Transporter associated with antigen processing
TCR	T cell receptor
Th	T follicular helper cell
THP 1	Human monocyte cell line
TLR	Toll-like receptor
TNF-α	Tumor necrosis factor alpha
TOX	Thymocyte selection associated high mobility group box protein
UL	Unique long region
US	Unique short region
vICA	Viral inhibitor of caspase 8
vMIA	Viral mitochondrial inhibitor of apoptosis
XBP1	X-box binding protein 1
YY1	Ying Yang 1
